# Successful Surgical Treatment of a Spontaneous Rupture of the Left Iliac Vein: What Is the Optimal and Radical Treatment?

**DOI:** 10.3400/avd.cr.25-00065

**Published:** 2025-11-14

**Authors:** Kei Morioka, Masanori Hirota, Shingo Kasahara

**Affiliations:** 1 Department of Cardiovascular Surgery, Okayama University Hospital, Okayama, Okayama, Japan; 2 Department of Cardiovascular Surgery, Showa Medical University Fujigaoka Hospital, Yokohama, Kanagawa, Japan

**Keywords:** spontaneous rupture of the iliac vein, deep vein thrombosis, arteriovenous shunt

## Abstract

Spontaneous rupture of the iliac vein (SRIV) requires surgical hemostasis and venous return restoration. We herein report a case treated with initial thrombus removal and direct venous repair. Because of early occlusion, a 2nd surgery was performed for iliac vein reconstruction using a 14-mm ringed Gore-Tex graft (W. L. Gore & Associates, Newark, DE, USA), and a 4-mm Gore-Tex arteriovenous shunt was created between the femoral artery and the femoral vein to prevent reocclusion. The patient had an uneventful recovery without recurrence. A single-stage procedure including hemostasis, vein replacement, and arteriovenous bypass may be ideal for radical SRIV treatment.

## Introduction

Spontaneous rupture of the iliac vein (SRIV) is an extremely rare disease requiring emergency surgical intervention owing to its highly lethal nature. A possible mechanism is closely related to the acute elevation of iliac venous pressure secondary to mechanical stress such as squatting.^1)^ Congestive blood flow in the iliac vein is also potentially responsible for SRIV.^1)^ Anatomically, compression of the left common iliac vein by the overlying right iliac artery and the presence of intraluminal fibrous membranes, commonly referred to as ‘‘web,” can impede venous return and predispose to rupture.^2)^ Although the primary objective in the treatment of SRIV is surgical hemostasis, restoration of venous return is important for radical treatment.

We herein report a case of a patient with SRIV who underwent radical treatment. Emergency surgery involved direct hemostasis and thrombectomy of the occluded left iliac vein. Because of postoperative reocclusion, the diseased left iliac vein with a web was excised and then reconstructed using a ringed Gore-Tex graft. To prevent reocclusion, an arteriovenous shunt was created in the left groin. In this report, we discuss the optimal surgical strategies for minimizing the need for reoperation.

## Case Report

**Fig. 1:**
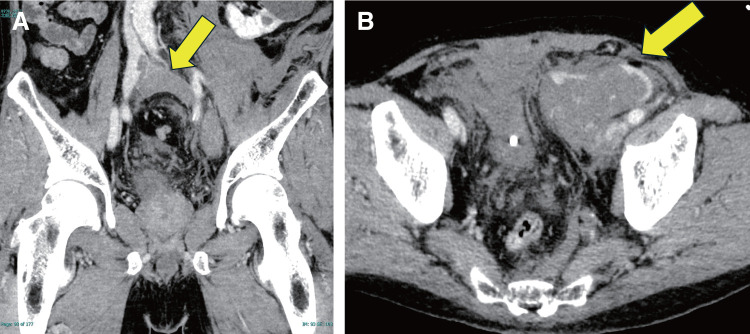
Computed tomography scans before surgery. (**A**) Venous thrombosis in the left iliac vein (arrow). (**B**) Hemorrhage in the retroperitoneal space (arrow).

A 69-year-old man was brought to the emergency department of another hospital in shock (systolic blood pressure, 50 mmHg). Computed tomography (CT) revealed thrombotic occlusion extending from the left common iliac vein to the left common femoral vein, along with rupture of the left external iliac vein (**Figs. 1A** and **1B**).

The patient was immediately transferred to our hospital for emergency surgical intervention. Prior to surgery, an inferior vena cava filter (Neuhaus Protect SE; Toray Medical, Tokyo, Japan) was positioned just distal to the renal veins, and an occlusion balloon catheter (Coda 12 Fr; Cook Medical, Bloomington, IN, USA) was placed in the inferior vena cava distal to the filter to prevent pulmonary emboli.

**Fig. 2:**
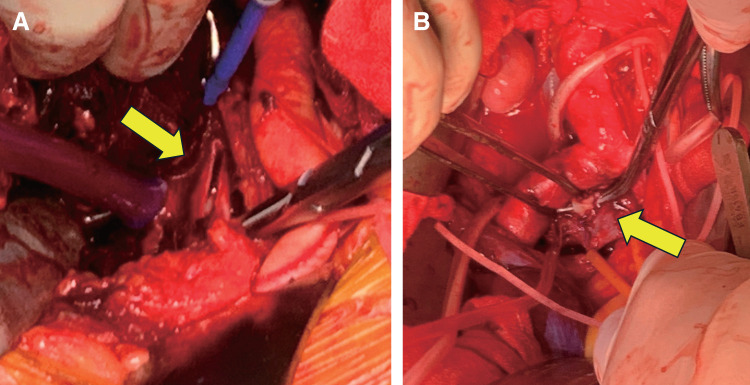
(**A**) Tear in the left iliac vein (arrow). (**B**) Intraluminal web structure in the left iliac vein (arrow).

After midline abdominal laparotomy, an additional oblique incision was made to expose the left iliac vein using a retroperitoneal approach. The large retroperitoneal hematoma was removed, and a 30-mm longitudinal tear was detected in the left external iliac vein (**Fig. 2A**). The large thrombus was removed using a Fogarty catheter (Edwards Lifesciences, Irvine, CA, USA). However, insufficient backflow from the proximal left iliac vein suggested a residual thrombus. Catheter advancement was obstructed at the right common iliac artery level. Therefore, the left iliac vein was opened. A web-like intraluminal structure causing the luminal stenosis was identified and completely excised (**Fig. 2B**). After achieving satisfactory liminal patency, the vein was closed. Successful venous return was verified using venography.

Despite systemic heparinization after surgery, the swelling in the left lower extremity worsened on postoperative day 1. Contrast-enhanced CT revealed a recurrent thrombotic occlusion extending from the left common iliac vein to the left common femoral vein. Emergency reoperation was performed to restore venous return.

**Fig. 3:**
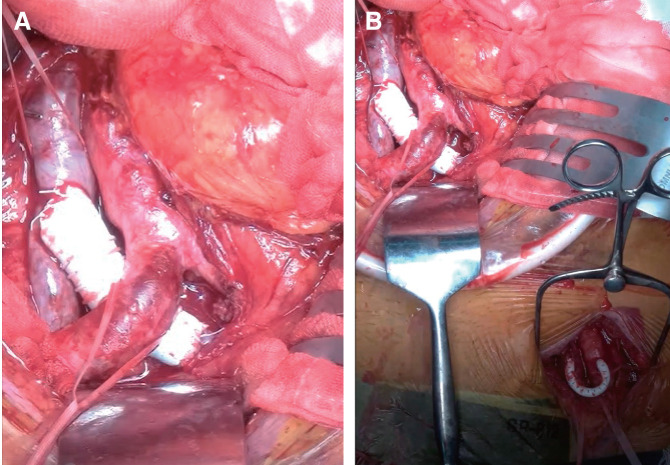
(**A**) Reconstruction of the left common iliac vein using a Gore-Tex graft (W. L. Gore & Associates, Newark, DE, USA) during the 2nd surgery. (**B**) Arteriovenous shunt created between the left femoral artery and the left femoral vein with a Gore-Tex graft.

Under general anesthesia, the abdomen was reopened. The inferior vena cava was clamped proximal to the confluence of the common iliac veins to prevent thrombus migration, after which the thrombus was removed with a Fogarty catheter. Both the rough intraluminal surface remaining after the resection of the web and the mild compression by the right iliac artery contributed to the reocclusion. The left common iliac vein was partially excised and replaced with a 14-mm ringed Gore-Tex prosthetic graft (W. L. Gore & Associates, Newark, DE, USA) (**Fig. 3A**). To prevent reocclusion, a 4-mm Gore-Tex arteriovenous shunt was created between the left femoral artery and the left femoral vein (**Fig. 3B**). Heparin administration was immediately restarted.

Laboratory tests revealed no abnormalities in protein C or S antibodies. After the withdrawal of heparin, warfarin and aspirin were initiated to prevent thrombus formation. Pathological examination of the left iliac vein wall revealed a mild partial fibrous intimal thickening without signs of inflammation or structural loss.

The patient was transferred to a rehabilitation facility on the 21st postoperative day under stable clinical conditions. After 2.6 years, the patient was doing well and had no left leg edema.

## Discussion

In this report, the patient with SRIV was initially treated through restoration of hemostasis, which contributed to immediate recovery from hemodynamic instability. However, despite the removal of the responsible intraluminal structure in the left iliac vein, venous return was obstructed because of thrombus formation during heparinization. A second procedure was required to restore venous return. The obstructed left common iliac vein was excised and replaced with a 14-mm ringed Gore-Tex prosthetic graft. To prevent reocclusion, a 4-mm Gore-Tex arteriovenous shunt was created in the groin. Although we performed 2 surgeries, the patient was doing well without blue phlegmasia. Accordingly, our surgical strategy is radical and satisfactory for patients with SRIV.

Currently, there are no standardized guidelines or consensus regarding SRIV treatment. The reported clinical management strategies include invasive operations,^2–5)^ catheter-based interventions,^6,7)^ and conservative treatments.^8,9)^ Although both invasive surgeries and catheter-based interventions can contribute to rapid recovery from hemodynamic instability, conservative treatment carries the risk of reocclusion, lower extremity edema, and re-rupture. Direct hemostasis should be the primary objective for SRIV, and the prevention of reocclusion of the left iliac vein should be the secondary objective to prevent blue phlegmasia. The venous lesion was located immediately under the right iliac artery.^1)^ Therefore, we recommend a single surgery combining direct hemostasis and graft replacement as the optimal and radical treatment.

In cases of SRIV, reocclusion of the left iliac vein leads to a poor prognosis; severe edema of the thigh and lower leg may lead to limb amputation and decreased quality of life. ^2,7,10)^

In this case, we selected an artificial graft for the arteriovenous shunt because the venous return was obstructed after harvesting the autologous saphenous vein. Accordingly, an additional arteriovenous shunt with an artificial graft would be advantageous for preventing blue phlegmasia secondary to obstruction of the venous return.

We successfully treated a patient with SRIV through 2 surgeries, and the postoperative course was satisfactory. However, a single combined operation that addresses the hemodynamic instability and the diseased left iliac vein would be optimal and radical for such patients. An additional arteriovenous shunt with an artificial graft is recommended for 1-stage operations. Postoperative blue phlegmasia is a critical complication of SRIV, and intensive treatments should be performed during a single operation.

## Conclusion

Surgical management of SRIV is highly intensive, and the prevention of postoperative blue phlegmasia is critical for improving outcomes. Restoration of hemostasis, venous reconstruction, and arteriovenous shunting during the initial surgery can significantly contribute to optimal clinical results. To achieve the 2 essential objectives of immediate hemostasis and prevention of venous obstruction, a comprehensive, well-planned, single-stage surgery is ideal.

### Additional Remarks

This study was presented at the 51st Annual Meeting of the Japanese Society for Vascular Surgery (Tokyo, Japan, June 1, 2023).
